# Supplementary material for: Male fertility thermal limits predict vulnerability to climate warming
Source: Nat Commun. 2021 Apr 13;12:2214. doi: 10.1038/s41467-021-22546-w (PMC8044094; doi:10.1038/s41467-021-22546-w)
Supplement: Supplementary file 1 — Supplementary Information [file 41467_2021_22546_MOESM1_ESM.pdf]

**Supplementary Information**

# Male fertility thermal limits predict vulnerability to climate warming

**Belinda van Heerwaarden and Carla M. Sgrò**

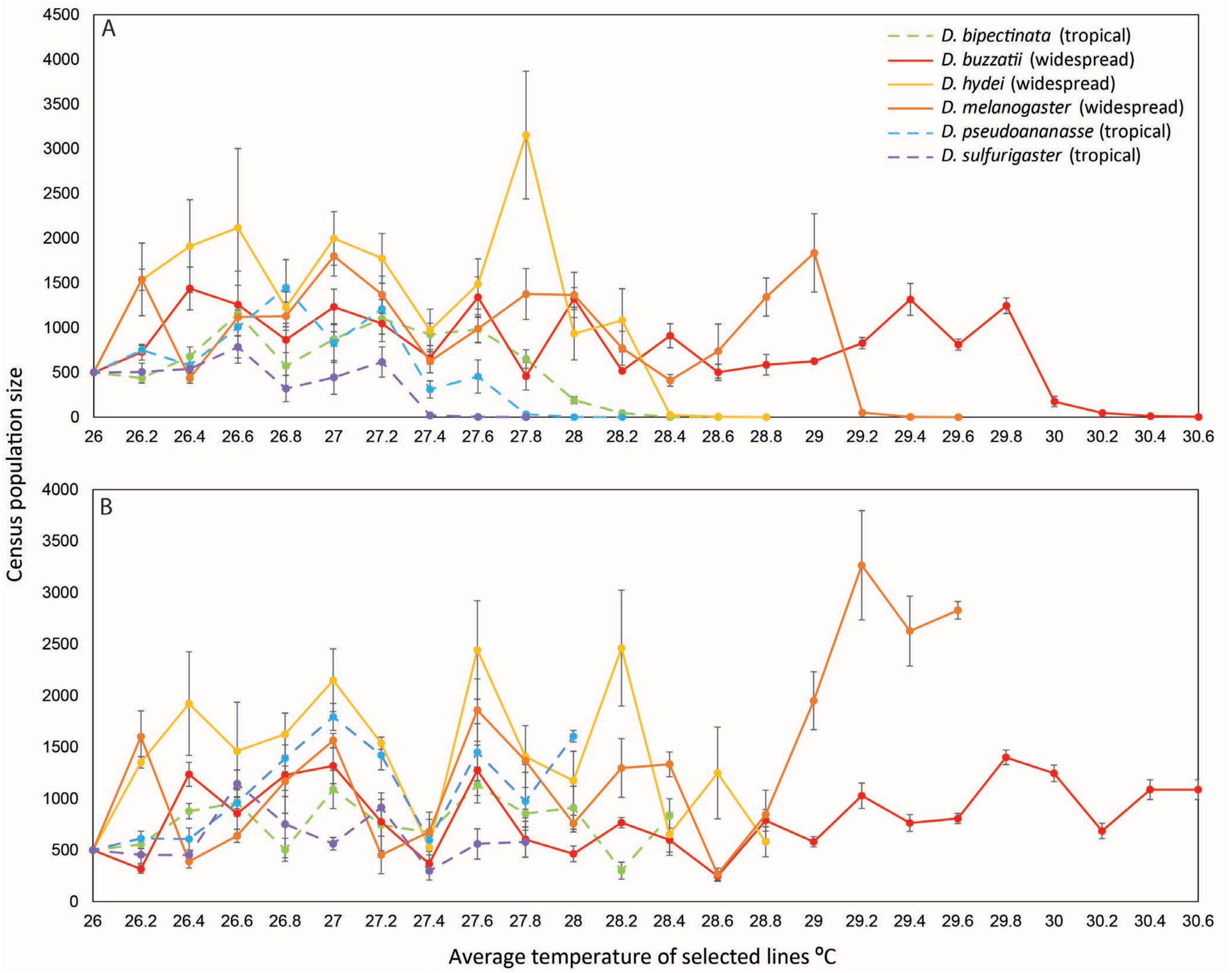


Figure S1. Changes in population size in selected (A) and control lines (B) in tropical (dashed lines) and widespread (solid lines) *Drosophila* species. Four replicate selection lines of *D. bipectinata* (tropical)*, D. buzzatii* (widespread cactophilic)*, D. hydei* (widespread)*, D. melanogaster* (widespread)*, D. pseudoananassae* (tropical) and *D. sulfurigaster* (tropical) were initiated under a fluctuating temperature regime averaging 26 ºC (± 3 ºC) and exposed to a 0.2 ºC increase in average temperature every two weeks (approximately one generation). Census population size (± SEM) was estimated at the end of each two-week warming period by assessing the census population size of each replicate line. The source data is provided in the Source data file.

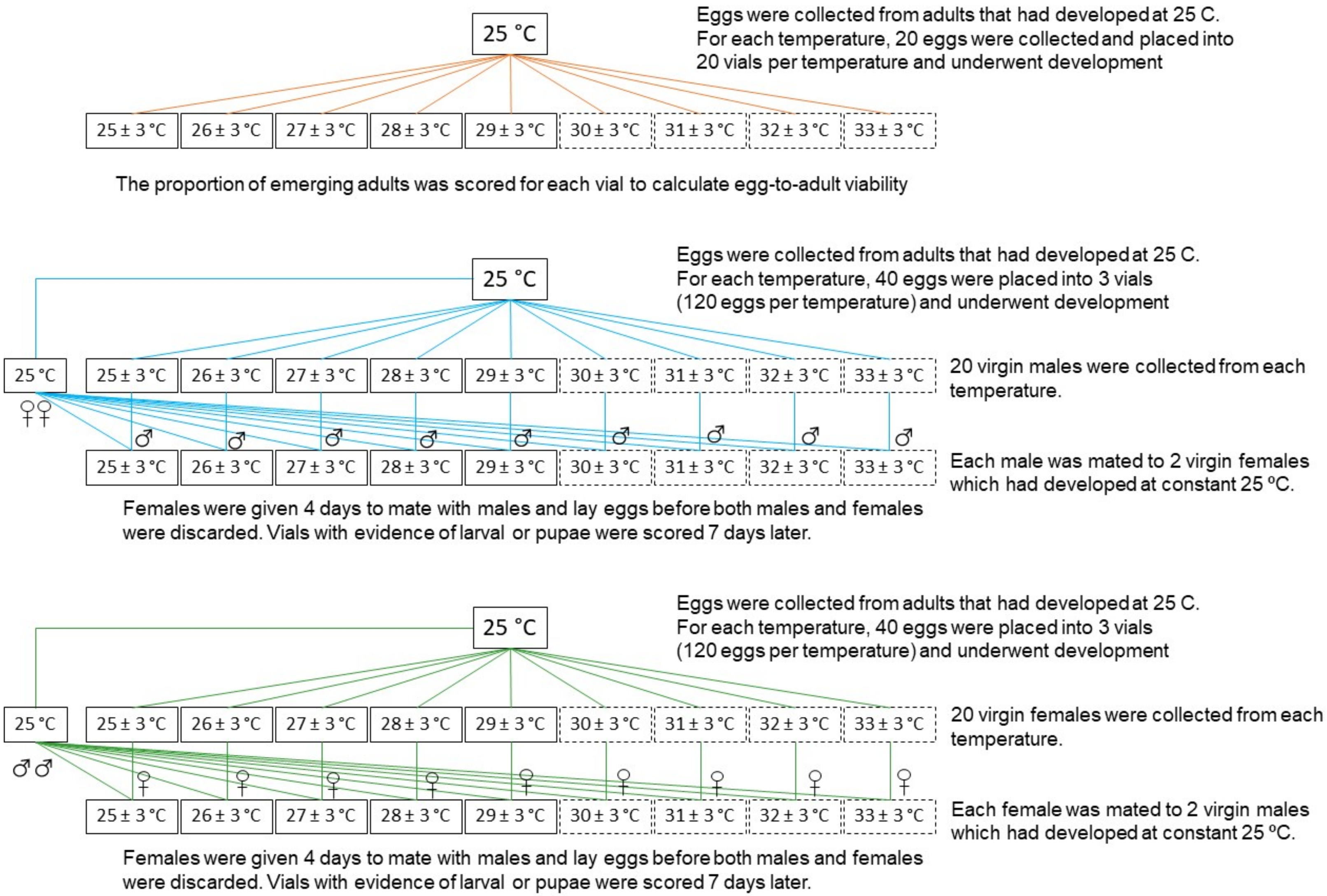


Figure S2 Initial trait assessment experimental design

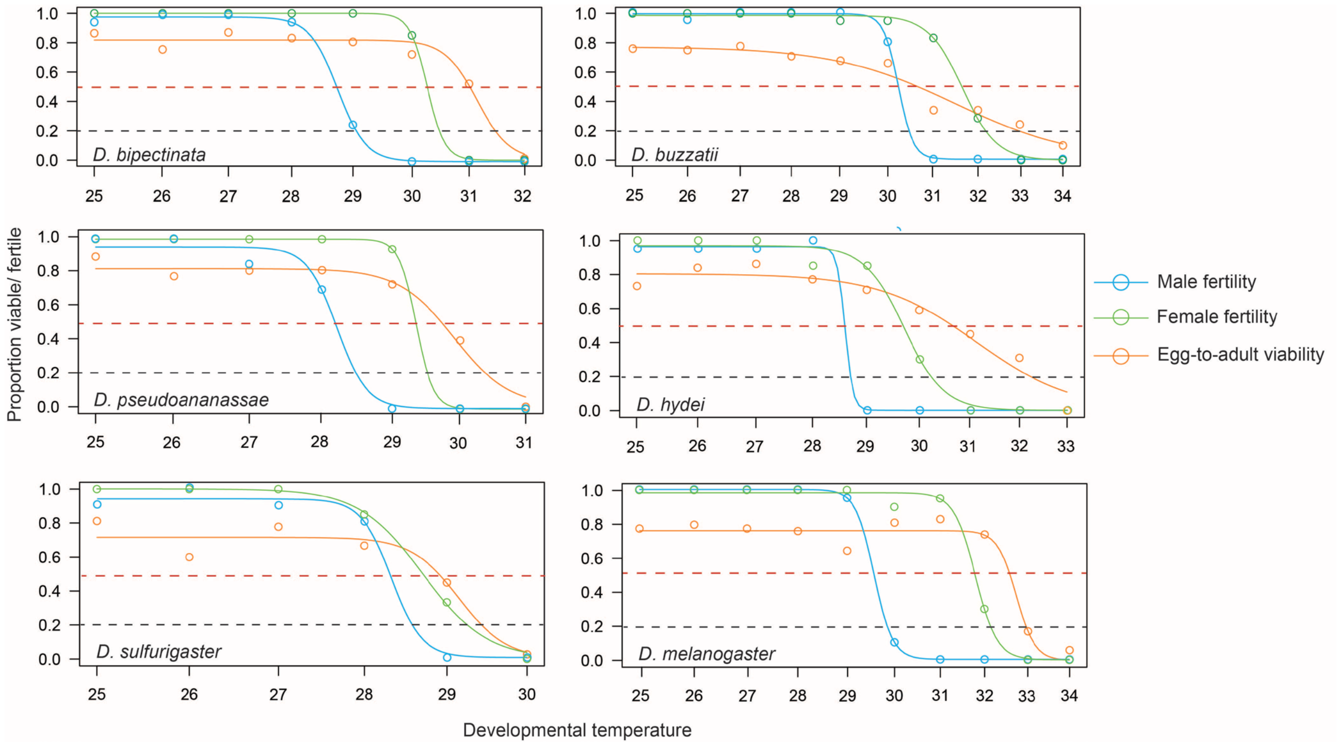


Figure S3. The relationship between mean developmental temperature (ºC) and mean male fertility (blue), female fertility (green) and egg-to-adult viability (orange) for *D. bipectinata* (tropical), *D. buzzatii* (widespread cactophilic), *D. pseudoananassae* (tropical), *D. hydei* (widespread), *D. sulfurigaster* (tropical) and *D. melanogaster* (widespread). The solid lines represent the fitted three-parameter log-logistic dose-response model. The dashed lines indicate where fertility/viability is 50% (red) and 20% (black). Note different scales on the x- axis. Source data are provided as a Source Data file.

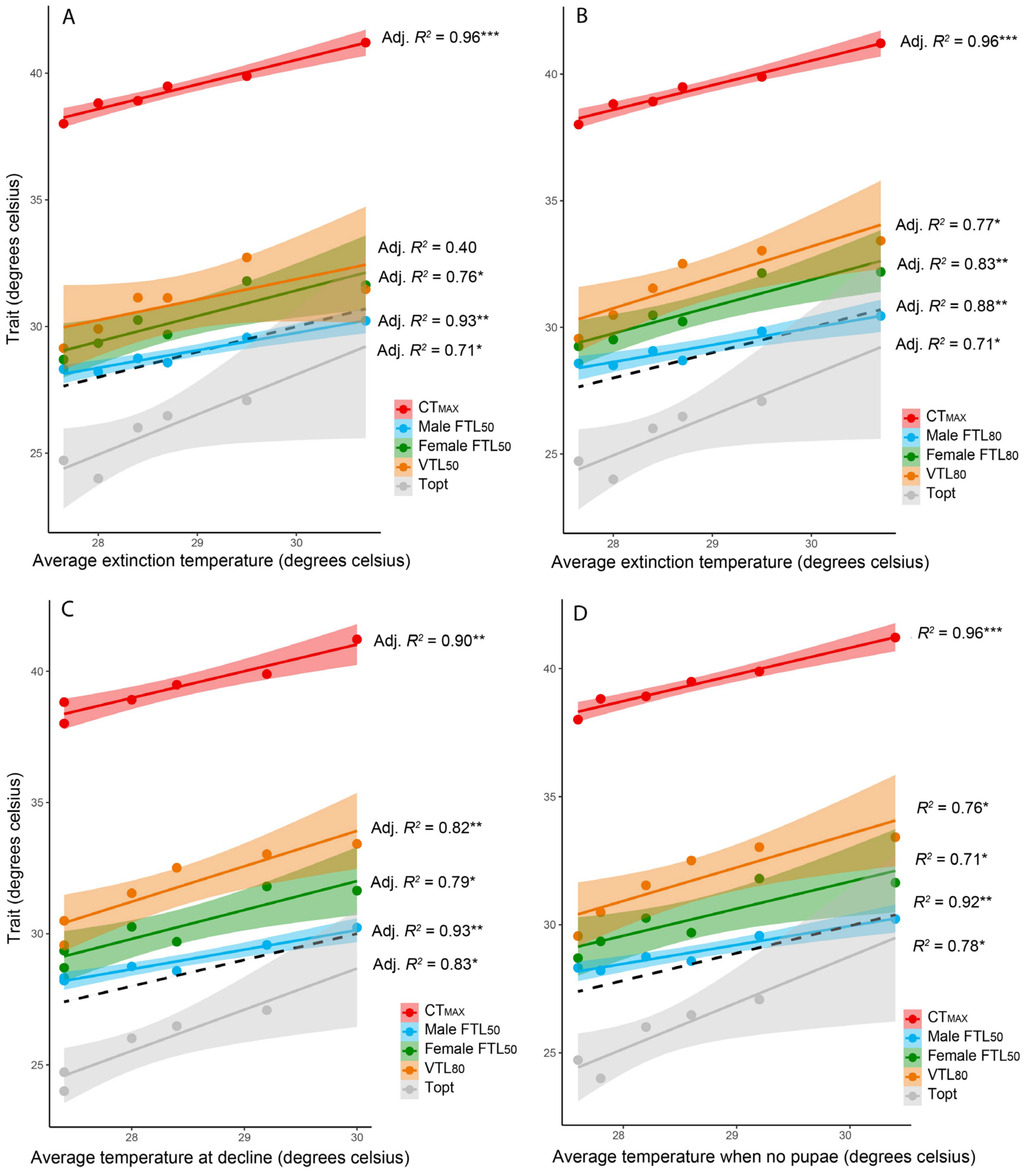


Figure S4. Associations between different estimates of thermal tolerance and average extinction temperature (A, B), average temperature at decline (C) and average temperature when there are no longer pupae (D) across 6 *Drosophila* species. Each point represents mean thermal tolerance for each species, the solid lines represent the fitted linear models and the shaded areas are the 95% confidence interval of these models. The dashed black line represents the direct relationship (slope = 1) between tolerance and average extinction (A/B), decline (C), no pupae temperature (D). The source data is provided in the Source data file.

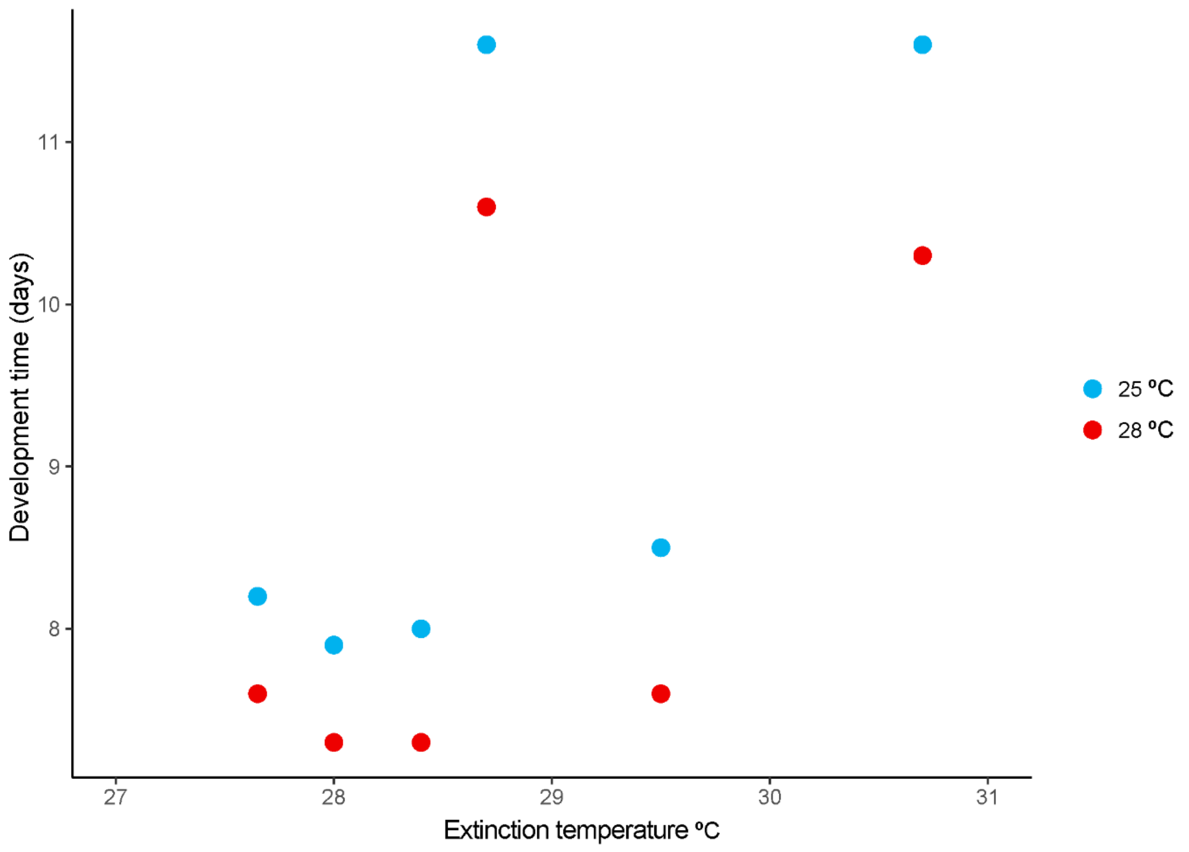


Figure S5. The relationship between average egg-to-adult development time at fluctuating 25 ºC (blue) and 28 ºC (red) and average extinction temperature. Each point represents the mean development time for each single species. See Table S2 for temperature regimes. The source data is provided in the Source data file.

Table S1. Collection information and details on the number of iso-female lines used to establish mass-bred populations for each species, the number of generations populations were maintained in the laboratory before the trait assessment or warming evolution experiment was commenced and the average development time from egg to adult at fluctuating 25 and 28 ºC (see Table S2).

| Species (species subgroup) | Distribution (tropical/ widespread) | Collection location (latitude, longitude) | Number of lines | Generations after collection (initial assessment/ warming experiment) | Development time at 25º C / 28º C (days) |
|---|---|---|---|---|---|
| *D. bipectinata* (ananassae) | tropical | 18.21 S, 145.78 E | 20 | 10 / 11 | 8.0 / 7.3 |
| *D. birchii* (montium) | tropical | 18.21 S, 145.78 E | 20 | 15 / NA | NA |
| *D. bunnanda* (montium) | tropical | 18.21 S, 145.78 E | 10 | 15 / NA | NA |
| *D. buzzatii* (mulleri) | widespread | 37.65 S, 144.77 E | 15 | 7 / 8 | 11.6 / 10.3 |
| *D. hydei* (hydei) | widespread | 37.80 S, 145.00 E | 20 | 5 / 6 | 11.6 / 10.6 |
| *D. melanogaster* (melanogaster) | widespread | 37.82 S, 145.02 E | 20 | 13 / 14 | 8.5 / 7.6 |
| *D. pseudoananassae* (ananassae) | tropical | 18.21 S, 145.78 E | 20 | 9 / 10 | 7.9 / 7.3 |
| *D. serrata* (montium) | widespread | 18.21 S, 145.78 E | 15 | 15 / NA | NA |
| *D. simulans* (melanogaster) | widespread | 37.82 S, 145.02 E | 20 | 14 / NA | NA |
| *D. sulfurigaster* (nasuta) | tropical | 18.21 S, 145.78 E | 20 | 10 / 11 | 8.2 / 7.6 |

Table S2 Temperature regime of cabinets. Relative humidity was kept constant at 90%.

| Lights | Time | Control Regime | Selection + 1.2 ºC | Selection + 2.2 ºC | Selection + 3.4 ºC | Selection + 4.2 ºC |
|---|---|---|---|---|---|---|
| 0 | 0:00 | 24 ºC | 25.2 ºC | 26.2 ºC | 27.4 ºC | 28.2 ºC |
| 0 | 3:00 | 23.5 ºC | 24.7 ºC | 25.7 ºC | 26.9 ºC | 27.7 ºC |
| 1 | 6:00 | 24.5 ºC | 25.7 ºC | 26.7 ºC | 27.9 ºC | 28.7 ºC |
| 1 | 7:00 | 26 ºC | 27.2 ºC | 28.2 ºC | 29.4 ºC | 30.2 ºC |
| 1 | 8:00 | 27 ºC | 28.2 ºC | 29.2 ºC | 30.4 ºC | 31.2 ºC |
| 1 | 9:00 | 28 ºC | 29.2 ºC | 30.2 ºC | 31.4 ºC | 32.2 ºC |
| 1 | 11:00 | 29 ºC | 30.2 ºC | 31.2 ºC | 32.4 ºC | 33.2 ºC |
| 1 | 15:00 | 28 ºC | 29.2 ºC | 30.2 ºC | 31.4 ºC | 32.2 ºC |
| 1 | 17:00 | 27 ºC | 28.2 ºC | 29.2 ºC | 30.4 ºC | 31.2 ºC |
| 0 | 18:00 | 26 ºC | 27.2 ºC | 28.2 ºC | 29.4 ºC | 30.2 ºC |
| 0 | 20:00 | 25 ºC | 26.2 ºC | 27.2 ºC | 28.4 ºC | 29.2 ºC |
| 0 | 23:00 | 24 ºC | 25.2 ºC | 26.2 ºC | 27.4 ºC | 28.2 ºC |
| | Average | 26 ºC | 27.2 ºC | 28.2 ºC | 29.4 ºC | 30.2 ºC |
| | Maximum | 29 ºC | 30.2 ºC | 31.2 ºC | 32.4 ºC | 33.2 ºC |

Table S3 Temperatures when species subjected to experimental warming began to decline, became sterile (no pupae) and went extinct, averaged across four replicate lines.

| Species | Average temperature at decline | Average temperature when sterile | Average temperature at extinction | Minimum temperature at extinction | Maximum temperature at extinction |
|---|---|---|---|---|---|
| *D. bipectinata* (T) | 28.0 ºC | 28.15 ºC | 28.4 ºC | 25.9 ºC | 31.4 ºC |
| *D. buzzatii* (W) | 30.0 ºC | 30.25 ºC | 30.7 ºC | 28.2 ºC | 33.7 ºC |
| *D. hydei* (W) | 28.4 ºC | 28.60 ºC | 28.7 ºC | 26.2 ºC | 31.7 ºC |
| *D. melanogaster* (W) | 29.2 ºC | 29.20 ºC | 29.5 ºC | 27.0 ºC | 32.5 ºC |
| *D. pseudoananassae* (T) | 27.4 ºC | 27.80 ºC | 28.0 ºC | 25.5 ºC | 31.0 ºC |
| *D. sulfurigaster* (T) | 27.4 ºC | 27.60 ºC | 27.65 ºC | 25.15 ºC | 30.65 ºC |

T = tropical species, W = widespread species

Table S4 Correlations (pairwise, two-sided Pearson) between traits across six species (Five species for comparisons with *T*opt. Significant correlations (*P* < 0.05, uncorrected) are highlighted in bold. Source data are provided as a Source Data file.

| Trait | | CTmax | Male $FTL_{50}$ | Female $FTL_{50}$ | $VTL_{80}$ |
|---|---|---|---|---|---|
| Male $FTL_{50}$ | Correlation | 0.917 | | | |
| | *P* | **0.01** | | | |
| | df | 4 | | | |
| Female $FTL_{50}$ | Correlation | 0.857 | 0.923 | | |
| | *P* | **0.03** | **0.01** | | |
| | df | 4 | 4 | | |
| $VTL_{80}$ | Correlation | 0.915 | 0.835 | 0.887 | |
| | *P* | **0.01** | **0.04** | **0.02** | |
| | df | 4 | 4 | 4 | |
| *T*opt | Correlation | 0.79 | 0.85 | 0.79 | 0.90 |
| | *P* | 0.11 | 0.07 | 0.11 | **0.04** |
| | df | 3 | 3 | 3 | 3 |

Table S5. Results from a paired *t-test* (two tailed) examining mean differences between average temperature at extinction, maximum temperature at extinction, average temperature when the population size of selected lines was significantly lower than control lines (decline temperature) and average temperature when pupae were no longer present in selected lines (sterile temperature) and thermal tolerance traits CTmax, $VTL_{80}$, male $FTL_{50}$, female $FTL_{50}$ and *T*opt across the six species exposed to experimental warming. Source data are provided as a Source Data file.

| Trait 1 | Trait 2 | Mean difference | *t* | df | *P* |
|---|---|---|---|---|---|
| Average extinction temperature | CTmax | -10.56 | -132.05 | 5 | <0.001 |
| | $VTL_{80}$ | -2.93 | -10.25 | 5 | <0.001 |
| | Male $FTL_{50}$ | -0.12 | -0.74 | 5 | 0.494 |
| | Female $FTL_{50}$ | -1.42 | -6.28 | 5 | 0.002 |
| | *T*opt | 2.79 | 8.61 | 4 | 0.001 |
| Maximum extinction temperature | CTmax | -7.56 | -94.55 | 5 | <0.001 |
| Average decline temperature | CTmax | -10.99 | -86.50 | 5 | <0.001 |
| | $VTL_{80}$ | -3.36 | -12.04 | 5 | <0.001 |
| | Male $FTL_{50}$ | -0.54 | -4.14 | 5 | 0.008 |
| | Female $FTL_{50}$ | -1.84 | -8.52 | 5 | <0.001 |
| | *T*opt | 2.42 | 8.69 | 4 | <0.001 |
| Average sterile temperature (no pupae) | CTmax | -10.76 | -126.82 | 5 | <0.001 |
| | $VTL_{80}$ | -3.13 | -10.38 | 5 | <0.001 |
| | Male $FTL_{50}$ | -0.31 | -2.23 | 5 | 0.076 |
| | Female $FTL_{50}$ | -1.61 | -6.46 | 5 | 0.001 |
| | *T*opt | 2.62 | 8.01 | 4 | 0.001 |

Table S6. Results from generalized linear mixed models (binomial distribution, logit link function) assessing the effect of treatment (control or selection) and temperature (control or selected (warmer) developmental temperatures) on male sterility on species that had undergone selection for experimental warming tolerance. Replicate lines are nested within treatment as a random effect. Source data are provided as a Source Data file.

| Warming | Species | Predictor | Estimate ± SE | df | $x^2$ | $P$ | Semi-partial $R^2$ (CI) |
|---|---|---|---|---|---|---|---|
| 1.2 ºC | *D. bipectinata* | Intercept | 0.868 ± 0.208 | 1 | 17.364 | <0.001 | |
| | | temperature | 0.413 ± 0.203 | 1 | 4.135 | **0.042** | 0.032 (0.00-0.12) |
| | | treatment | 0.009 ± 0.204 | 1 | 0.002 | 0.966 | <0.001 (0.00-0.04) |
| | | temp*treat | -0.083 ± 0.208 | 1 | 0.170 | 0.680 | 0.001 (0.00-0.05) |
| | | Rep (intercept) | Variance ± SD = 0.008 ± 0.088 | | | | |
| | *D. buzzatii* | Intercept | 1.947 ± 0.316 | 1 | 37.997 | <0.001 | |
| | | temperature | -0.143 ± 0.240 | 1 | 0.355 | 0.552 | 0.002 (0.00-0.04) |
| | | treatment | -0.177 ± 0.261 | 1 | 0.372 | 0.542 | 0.003 (0.00-0.05) |
| | | temp*treat | -0.014 ± 0.240 | 1 | 0.004 | 0.953 | <0.001 (0.00-0.03) |
| | | Rep (intercept) | Variance ± SD = 0.203 ± 0.451 | | | | |
| | *D. hydei* | Intercept | 1.881 ± 0.243 | 1 | 60.038 | <0.001 | |
| | | temperature | -0.320 ± 0.243 | 1 | 1.743 | 0.187 | 0.011 (0.00-0.07) |
| | | treatment | 0.041 ± 0.243 | 1 | 0.028 | 0.867 | <0.001 (0.00-0.03) |
| | | temp*treat | -0.215 ± 0.243 | 1 | 0.783 | 0.376 | 0.005 (0.00-0.05) |
| | | Rep (intercept) | Variance ± SD = <0.001 ± <0.001 | | | | |
| | *D. melanogaster* | Intercept | 3.290 ± 0.458 | 1 | 51.611 | <0.001 | |
| | | temperature | -0.374 ± 0.458 | 1 | 0.665 | 0.415 | 0.004 (0.00-0.05) |
| | | treatment | 0.374 ± 0.458 | 1 | 0.665 | 0.415 | 0.004 (0.00-0.05) |
| | | temp*treat | 0.374 ± 0.458 | 1 | 0.665 | 0.415 | 0.004 (0.00-0.05) |
| | | Rep (intercept) | Variance ± SD = <0.001 ± <0.001 | | | | |
| | *D. pseudoananassae* | Intercept | -0.171 ± 0.424 | 1 | 0.163 | 0.687 | |
| | | temperature | 0.666 ± 0.193 | 1 | 11.898 | **<0.001** | 0.054 (0.01-0.14) |
| | | treatment | 0.177 ± 0.425 | 1 | 0.174 | 0.676 | 0.004 (0.00-0.05) |
| | | temp*treat | 0.201 ± 0.191 | 1 | 1.101 | 0.294 | 0.006 (0.00-0.05) |
| | | Rep (intercept) | Variance ± SD = 1.155 ± 1.075 | | | | |
| 2.2 ºC | *D. buzzatii* | Intercept | 2.584 ± 0.411 | 1 | 39.628 | <0.001 | |
| | | temperature | 0.376 ± 0.342 | 1 | 1.214 | 0.271 | 0.008 (0.00-0.06) |
| | | treatment | 0.368 ± 0.363 | 1 | 1.024 | 0.312 | 0.010 (0.00-0.06) |
| | | temp*treat | 0.361 ± 0.341 | 1 | 1.118 | 0.290 | 0.007 (0.00-0.06) |
| | | Rep (intercept) | Variance ± SD = 0.098 ± 0.313 | | | | |
| | *D. melanogaster* | Intercept | 3.208 ± 0.534 | 1 | 36.097 | <0.001 | |
| | | temperature | 0.004 ± 0.419 | 1 | <0.001 | 0.993 | <0.001 (0.00-0.03) |
| | | treatment | 0.576 ± 0.467 | 1 | 1.520 | 0.218 | 0.011 (0.00-0.07) |
| | | temp*treat | -0.018 ± 0.419 | 1 | 0.002 | 0.965 | <0.001 (0.00-0.03) |
| | | Rep (intercept) | Variance ± SD = 0.316 ± 0.562 | | | | |
| 3.4 ºC | *D. buzzatii* | Intercept | 2.784 ± 0.419 | 1 | 44.101 | <0.001 | |
| | | temperature | 0.156 ± 0.419 | 1 | 0.138 | 0.710 | 0.001 (0.00-0.05) |
| | | treatment | -0.082 ± 0.419 | 1 | 0.038 | 0.845 | <0.001 (0.00-0.04) |
| | | temp*treat | 0.509 ± 0.419 | 1 | 1.476 | 0.224 | 0.012 (0.00-0.08) |
| | | Rep (intercept) | Variance ± SD = <0.001 ± <0.001 | | | | |
| 4.2 ºC | *D. buzzatii* | Intercept | 0.457 ± 0.368 | 1 | 1.532 | 0.216 | |
| | | temperature | 1.752 ± 0.383 | 1 | 20.934 | **<0.001** | 0.259 (0.141-0.39) |
| | | treatment | -0.427 ± 0.367 | 1 | 1.355 | 0.244 | 0.022 (0.000-0.10) |
| | | temp*treat | -0.588 ± 0.350 | 1 | 2.828 | 0.093 | 0.046 (0.002-0.14) |
| | | Rep (intercept) | Variance ± SD = 0.181 ± 0.425 | | | | |

Significant *p*-values (α = 0.05) for fixed effects are in bold.

Table S7. Results from linear mixed models (gaussian distribution, identity link function) assessing the effect of treatment (control or selection) and temperature (control or selected (warmer) developmental temperatures) on male fertility on species that had undergone selection for experimental warming tolerance. Replicate lines are nested within treatment as a random effect. Source data are provided as a Source Data file.

| Warming | Species | Predictor | Estimate ± SE | df | $x^2$ | $P$ | Semi-partial $R^2$ (CI) |
|---|---|---|---|---|---|---|---|
| 1.2 ºC | *D. bipectinata* | Intercept | 8.054 ± 1.365 | 1 | 34.806 | <0.001 | |
| | | temperature | 1.174 ± 0.668 | 1 | 3.091 | 0.104 | 0.020 (0.00-0.09) |
| | | treatment | 0.159 ± 1.365 | 1 | 0.014 | 0.907 | <0.001 (0.00-0.04) |
| | | temp*treat | 0.240 ± 0.668 | 1 | 0.129 | 0.720 | 0.001 (0.00-0.04) |
| | | Rep (intercept) | Variance ± SD = 9.729 ± 3.119 | | | | |
| | *D. buzzatii* | Intercept | 18.368 ± 2.427 | 1 | 52.277 | <0.001 | |
| | | temperature | 0.103 ± 1.227 | 1 | 0.007 | 0.933 | <0.001 (0.00-0.03) |
| | | treatment | -2.013 ± 2.427 | 1 | 0.688 | 0.407 | 0.015 (0.00-0.08) |
| | | temp*treat | -2.808 ± 1.227 | 1 | 5.233 | **0.022** | 0.029 (0.001-0.1) |
| | | Rep (intercept) | Variance ± SD = 35.06 ± 5.92 | | | | |
| | *D. hydei* | Intercept | 25.013 ± 1.385 | 1 | 326.10 | <0.001 | |
| | | temperature | -2.238 ± 1.151 | 1 | 3.784 | 0.052 | 0.023 (0.00-0.09) |
| | | treatment | 0.326 ± 1.385 | 1 | 0.055 | 0.814 | <0.001 (0.00-0.03) |
| | | temp*treat | -1.276 ± 1.151 | 1 | 1.230 | 0.268 | 0.008 (0.00-0.06) |
| | | Rep (intercept) | Variance ± SD = 4.76 ± 2.18 | | | | |
| | *D. melanogaster* | Intercept | 28.271 ± 1.258 | 1 | 505.24 | <0.001 | |
| | | temperature | -5.420 ± 1.266 | 1 | 18.341 | **<0.001** | 0.119 (0.04-0.22) |
| | | treatment | 1.445 ± 1.356 | 1 | 1.135 | 0.287 | 0.010 (0.00-0.06) |
| | | temp*treat | 2.558 ± 1.266 | 1 | 4.084 | **0.043** | 0.030 (0.001-0.10) |
| | | Rep (intercept) | Variance ± SD = 1.90 ± 1.38 | | | | |
| | *D. pseudoananassae* | Intercept | 4.587 ± 0.995 | 1 | 21.239 | <0.001 | |
| | | temperature | 2.177 ± 0.477 | 1 | 20.881 | **<0.001** | 0.103 (0.03-0.20) |
| | | treatment | 1.749 ± 0.100 | 1 | 3.089 | 0.079 | 0.069 (0.01-0.16) |
| | | temp*treat | 1.465 ± 0.477 | 1 | 9.451 | **0.002** | 0.049 (0.01-0.13) |
| | | Rep (intercept) | Variance ± SD = 6.11 ± 2.47 | | | | |
| 2.2 ºC | *D. buzzatii* | Intercept | 48.743 ± 3.610 | 1 | 182.29 | <0.001 | |
| | | temperature | 4.980 ± 2.763 | 1 | 3.249 | 0.071 | 0.020 (0.00-0.08) |
| | | treatment | 1.500 ± 3.610 | 1 | 0.173 | 0.678 | 0.002 (0.00-0.04) |
| | | temp*treat | -0.177 ± 2.763 | 1 | 0.004 | 0.949 | <0.001 (0.00-0.03) |
| | | Rep (intercept) | Variance ± SD = 43.21 ± 6.57 | | | | |
| | *D. melanogaster* | Intercept | 43.679 ± 1.837 | 1 | 565.65 | <0.001 | |
| | | temperature | 1.616 ± 1.566 | 1 | 1.064 | 0.302 | 0.007 (0.00-0.06) |
| | | treatment | 0.141 ± 1.835 | 1 | 0.006 | 0.939 | <0.001 (0.00-0.03) |
| | | temp*treat | -0.115 ± 1.566 | 1 | 0.005 | 0.942 | <0.001 (0.00-0.03) |
| | | Rep (intercept) | Variance ± SD = 7.33 ± 2.71 | | | | |
| 3.4 ºC | *D. buzzatii* | Intercept | 29.052 ± 1.928 | 1 | 227.13 | <0.001 | |
| | | temperature | 1.336 ± 1.664 | 1 | 0.645 | 0.422 | 0.005 (0.00-0.06) |
| | | treatment | 0.882 ± 1.923 | 1 | 0.209 | 0.647 | 0.002 (0.00-0.05) |
| | | temp*treat | 1.031 ± 1.664 | 1 | 0.384 | 0.536 | 0.003 (0.00-0.05) |
| | | Rep (intercept) | Variance ± SD = 6.496 ± 2.549 | | | | |
| 4.2 ºC | *D. buzzatii* | Intercept | 29.409 ± 2.344 | 1 | 157.35 | <0.001 | |
| | | temperature | 25.979 ± 2.344 | 1 | 122.79 | **<0.001** | 0.512 (0.40-0.62) |
| | | treatment | -2.858 ± 2.344 | 1 | 1.486 | 0.223 | 0.012 (0.00-0.09) |
| | | temp*treat | -3.254± 2.344 | 1 | 1.926 | 0.165 | 0.016 (0.00-0.08) |
| | | Rep (intercept) | Variance ± SD = <0.001 ± 0.001 | | | | |

Significant *p*-values (α = 0.05) for fixed effects are in bold.

Table S8. Results from linear mixed models (gaussian distribution, identity link function) assessing the effect of treatment (control or selection) and temperature (control or selected (warmer) developmental temperatures) on CTmax on species that had undergone selection for experimental warming tolerance. Replicate lines are nested within treatment as a random effect. Source data are provided as a Source Data file.

| Warming | Species | Predictor | Estimate ± SE | df | $x^2$ | P | Semi-partial $R^2$ (CI) |
|---|---|---|---|---|---|---|---|
| 2.2 ºC | *D. buzzatii* | Intercept | 41.560 ± 0.034 | 1 | 1.4e6 | <0.001 | |
| | | temperature | -0.052 ± 0.034 | 1 | 2.339 | 0.126 | 0.007 (0.00-0.04) |
| | | treatment | -0.014 ± 0.034 | 1 | 0.164 | 0.685 | 0.001 (0.00-0.02) |
| | | temp*treat | -0.013 ± 0.034 | 1 | 0.138 | 0.710 | <0.001 (0.00-0.02) |
| | | Rep (intercept) | Variance ± SD = <0.001 ± <0.001 | | | | |
| | *D. melanogaster* | Intercept | 41.054 ± 0.012 | 1 | 1.2e7 | <0.001 | |
| | | temperature | -0.103 ± 0.012 | 1 | 60.94 | **<0.001** | 0.173 (0.11-0.25) |
| | | treatment | 0.005 ± 0.012 | 1 | 0.167 | 0.683 | 0.001 (0.00-0.02) |
| | | temp*treat | -0.007 ± 0.012 | 1 | 0.311 | 0.577 | 0.001 (0.00-0.02) |
| | | Rep (intercept) | Variance ± SD = <0.001 ± <0.001 | | | | |
| 3.4 ºC | *D. buzzatii* | Intercept | 41.570 ± 0.018 | 1 | 5.3e6 | <0.001 | |
| | | temperature | -0.081 ± 0.018 | 1 | 19.92 | **<0.001** | 0.078 (0.03-0.15) |
| | | treatment | 0.003 ± 0.018 | 1 | 0.021 | 0.884 | <0.001 (0.00-0.02) |
| | | temp*treat | 0.005 ± 0.018 | 1 | 0.083 | 0.774 | <0.001 (0.00-0.02) |
| | | Rep (intercept) | Variance ± SD = <0.001 ± 0.001 | | | | |
| 4.2 ºC | *D. buzzatii* | Intercept | 41.359 ± 0.021 | 1 | 4.0e6 | <0.001 | |
| | | treatment | -0.003 ± 0.021 | 1 | 0.015 | 0.903 | <0.001 (0.00-0.04) |
| | | Rep (intercept) | Variance ± SD = 0.002 ± 0.041 | | | | |

Significant *p*-values (α = 0.05) for fixed effects are in bold.

Table S9 Estimates of mean CTmax, male $FTL_{50}$, female $FTL_{50}$ and $VTL_{80}$ for the 10 species where these traits were assessed.

| Species | CTmax (±SEM) | Male $FTL_{50}$ (±SEM) | Female $FTL_{50}$ (±SEM) | $VTL_{80}$ (±SEM) |
|---|---|---|---|---|
| *D. bipectinata* | 38.92 ± 0.04 | 28.75 ± 0.09 | 30.26 ± 0.10 | 31.54 ± 0.25 |
| *D. birchii* | 38.11 ± 0.04 | 25.80 ± 0.22 | 27.99 ± 0.06 | NA |
| *D. bunnanda* | 38.42 ± 0.05 | 26.69 ± 0.16 | 28.08 ± 0.11 | NA |
| *D. buzzatii* | 41.21 ± 0.05 | 30.23 ± 0.11 | 31.64 ± 0.09 | 33.42 ± 0.37 |
| *D. hydei* | 39.49 ± 0.05 | 28.58 ± 0.34 | 29.69 ± 0.10 | 32.51 ± 0.26 |
| *D. melanogaster* | 39.89 ± 0.03 | 29.57 ± 0.07 | 31.8 ± 0.08 | 33.03 ± 0.09 |
| *D. pseudoananassae* | 38.82 ± 0.06 | 28.21 ± 0.09 | 29.35 ± 0.11 | 30.49 ± 0.15 |
| *D. serrata* | 38.61 ± 0.04 | 27.24 ± 0.08 | 28.47 ± 0.21 | NA |
| *D. simulans* | 38.93 ± 0.04 | 29.32 ± 0.13 | NA | NA |
| *D. sulfurigaster* | 38.01 ± 0.05 | 28.32 ± 0.12 | 28.7 ± 0.10 | 29.56 ± 0.15 |
