# Supplementary material for: Male fertility thermal limits predict vulnerability to climate warming
Source: Nat Commun. 2021 Apr 13;12:2214. doi: 10.1038/s41467-021-22546-w (PMC8044094; doi:10.1038/s41467-021-22546-w)
Supplement: Supplementary file 3 — Reporting Summary [file 41467_2021_22546_MOESM3_ESM.pdf]

nature research

Corresponding author(s): Belinda van Heerwaarden

Last updated by author(s): Mar 5, 2021

# Reporting Summary

Nature Research wishes to improve the reproducibility of the work that we publish. This form provides structure for consistency and transparency in reporting. For further information on Nature Research policies, see our Editorial Policies and the Editorial Policy Checklist.

## Statistics

For all statistical analyses, confirm that the following items are present in the figure legend, table legend, main text, or Methods section.

| n/a | Confirmed | |
|---|---|---|
| ☐ | ☒ | The exact sample size (*n*) for each experimental group/condition, given as a discrete number and unit of measurement |
| ☐ | ☒ | A statement on whether measurements were taken from distinct samples or whether the same sample was measured repeatedly |
| ☐ | ☒ | The statistical test(s) used AND whether they are one- or two-sided<br>*Only common tests should be described solely by name; describe more complex techniques in the Methods section.* |
| ☒ | ☐ | A description of all covariates tested |
| ☐ | ☒ | A description of any assumptions or corrections, such as tests of normality and adjustment for multiple comparisons |
| ☐ | ☒ | A full description of the statistical parameters including central tendency (e.g. means) or other basic estimates (e.g. regression coefficient) AND variation (e.g. standard deviation) or associated estimates of uncertainty (e.g. confidence intervals) |
| ☐ | ☒ | For null hypothesis testing, the test statistic (e.g. *F*, *t*, *r*) with confidence intervals, effect sizes, degrees of freedom and *P* value noted<br>*Give P values as exact values whenever suitable.* |
| ☒ | ☐ | For Bayesian analysis, information on the choice of priors and Markov chain Monte Carlo settings |
| ☒ | ☐ | For hierarchical and complex designs, identification of the appropriate level for tests and full reporting of outcomes |
| ☒ | ☐ | Estimates of effect sizes (e.g. Cohen's *d*, Pearson's *r*), indicating how they were calculated |

*Our web collection on statistics for biologists contains articles on many of the points above.*

## Software and code

Policy information about availability of computer code

| | |
|---|---|
| Data collection | No software was used |
| Data analysis | R version 3.6.3; drc package, version 3.0-1; lme4 package, version 1.2-21; car package, version 3.0-6; DHARMa package, version 0.2.7; lsmeans package, version 2.30-0, r2glmm package, version 0.1.2; |

For manuscripts utilizing custom algorithms or software that are central to the research but not yet described in published literature, software must be made available to editors and reviewers. We strongly encourage code deposition in a community repository (e.g. GitHub). See the Nature Research guidelines for submitting code & software for further information.

## Data

Policy information about availability of data

All manuscripts must include a data availability statement. This statement should provide the following information, where applicable:

- Accession codes, unique identifiers, or web links for publicly available datasets
- A list of figures that have associated raw data
- A description of any restrictions on data availability

Source data are provided with this paper (source_data.zip). Distribution data was extracted for each Drosophila species from the taxodros website https://www.taxodros.uzh.ch/) and environmental data for each distribution data point was extracted from the WorldClim dataset (https://www.worldclim.org).

# Field-specific reporting

Please select the one below that is the best fit for your research. If you are not sure, read the appropriate sections before making your selection.

☐ Life sciences ☐ Behavioural & social sciences ☒ Ecological, evolutionary & environmental sciences

For a reference copy of the document with all sections, see nature.com/documents/nr-reporting-summary-flat.pdf

# Ecological, evolutionary & environmental sciences study design

All studies must disclose on these points even when the disclosure is negative.

| | |
|---|---|
| Study description | This study subjected laboratory populations of 6 different species of Drosophila (3 widespread, 3 tropical) to two temperature treatments: one was maintained under controlled fluctuating temperatures ranging from 23.5 to 29 degrees Celsius, average 26 degrees, while the other temperature treatment involved experimental warming of 0.2 degrees Celsius every 2 weeks (~1 generation). Four biological replicates were used for each species/ temperature treatment. Male fertility/ sterility and CTmax was assessed for each replicate line after each ~1 degree of experimental warming in flies developing under control and 'selected' temperature conditions in a randomized factorial design within each temperature/ treatment.<br>Initial viability, male and female fertility and CTmax were assessed on these 6 species, as well as on 4 additional Drosophila species (2 tropical and 2 widespread - fertility and CTmax only). Viability, male and female fertility were estimated at 5- 9 different fluctuating temperatures, averages ranging from 25 - 33 degrees Celsius. |
| Research sample | This study uses laboratory populations of D. bipectinata, D. buzzatii, D. hydei, D. melanogaster, D. pseudoananassae, D. sulfurigaster, D. birchii, D. bunnanda, D. serrata and D. simulans recently collected from the field around Kirrama (tropical) and Melbourne (temperate), Australia. These species differ in their thermal tolerance and distribution across latitude and are suitable for addressing the research questions. CTmax was assessed on adult female flies (7 days old), viability was assessed as egg-to-adult viability (sex not determined), male and female fertility was measured over 4 days on adults (4 to 5 days old) after developing from egg to adults at different fluctuating temperatures. Topt was taken from the literature (one empirical study using different constant temperatures: MacLean et al 2019 https://doi.org/10.1098/rstb.2018.0548). |
| Sampling strategy | Unbiased and randomized sampling of individuals scored for traits. For initial trait assessments, eggs were picked prior to thermal tolerance assays to control larval density and random adults were chosen to assay. For cage assessments, random adult flies emerging from bottles were chosen to assay. No sample size calculation was carried out. We chose our sample sizes based on logistical constraints (see methods) and past experience (e.g. van Heerwaarden et al 2012, Journal of Evolutionary Biology; Sgro et al 2010 , Journal of Evolutionary Biology, van Heerwaarden and Sgro 2014, Proc R Soc B) |
| Data collection | Data was collected by the authors of this study and a research assistant. The extinction experiment was set up by van Heerwaarden and an RA by randomly allocating adult flies to control and selected replicate lines and placing them homemade plastic cages in temperature and humidity controlled cabinets (PHCbi). Flies were censored every 2 weeks by either van Heerwaarden or an RA, using a Drosophila Funnel Monitor (TriKinetics Inc). van Heerwaarden and an RA set up and scored all thermal tolerance traits. Fertility and viability were assessed using the same temperature/ humidity controlled cabinets and were scored (counted) by van Heerwaarden and the RA. Ctmax was assessed using a thermocycler and a water bath and scored by two experienced scorers (van Heerwaarden and Vanessa Kellermann). |
| Timing and spatial scale | Experimental evolution occurred from November 2017 to February 2019. Initial assessments were conducted in November 2017. Replicate line growth rates were assessed every 2 weeks from November 2017 to February 2019. Traits were assessed in replicate lines ~ every 12 weeks from November 2017 to February 2019. |
| Data exclusions | No data exclusion. |
| Reproducibility | It was not logistically feasible to repeat this experiment |
| Randomization | All flies were assigned to treatments randomly. Flies were moved around temperature cabinets every time a bottle was added (every 2- 3 days,to account for within cabinet temperature fluctuations. |
| Blinding | Lines were re-coded when scoring CTmax. Replicate control and selected lines were not recoded when censoring flies, as we did not want to mix up temperature treatments when placing them back at their respective cabinets. When assessing fertility and viability at under different temperature treatments, we did not recode vials as number of eclosing adults is not a subjective trait. |

Did the study involve field work? ☐ Yes ☒ No

# Reporting for specific materials, systems and methods

We require information from authors about some types of materials, experimental systems and methods used in many studies. Here, indicate whether each material, system or method listed is relevant to your study. If you are not sure if a list item applies to your research, read the appropriate section before selecting a response.

## Materials & experimental systems

| n/a | Involved in the study |
|---|---|
| ☒ | ☐ Antibodies |
| ☒ | ☐ Eukaryotic cell lines |
| ☒ | ☐ Palaeontology and archaeology |
| ☐ | ☒ Animals and other organisms |
| ☒ | ☐ Human research participants |
| ☒ | ☐ Clinical data |
| ☒ | ☐ Dual use research of concern |

## Methods

| n/a | Involved in the study |
|---|---|
| ☒ | ☐ ChIP-seq |
| ☒ | ☐ Flow cytometry |
| ☒ | ☐ MRI-based neuroimaging |

# Animals and other organisms

Policy information about studies involving animals; ARRIVE guidelines recommended for reporting animal research

| | |
|---|---|
| Laboratory animals | Drosophila bipectinata, Drosophila birchii, Drosophila bunnanda, Drosophila buzzatii, Drosophila hydei, Drosophila melanogaster, Drosophila pseudoananassae, Drosophila serrata, Drosophila simulans, Drosophila sulfurigaster, both sexes were used depending on the trait (see methods), traits were assessed on pre-adult and adult stages (see methods). |
| Wild animals | The study did not involve wild animals |
| Field-collected samples | Field collected populations were maintained as discrete generations, in large population sizes (>1000 individuals) on potato dextrose agar medium at a constant 25 ºC under a 12:12 light cycle. Populations of 4 species are still in the laboratory; the remaining 6 were lost after the experiment due to temperature cabinet failure. |
| Ethics oversight | No ethical approval required |

Note that full information on the approval of the study protocol must also be provided in the manuscript.
